# How to make green and purple from gold

**DOI:** 10.1093/plphys/kiad653

**Published:** 2023-12-09

**Authors:** Ryo Yokoyama

**Affiliations:** Assistant Features Editor, Plant Physiology, American Society of Plant Biologists; Max-Planck-Institute of Molecular Plant Physiology, Am Mühlenberg 1, Potsdam-Golm 14476, Germany

A diverse array of colorful plant pigments showcases a remarkable chemical diversity that prominently distinguishes plants from the animal kingdom. With their unique chemical properties, such as light absorption at specific wavelengths, these plant pigments play critical roles in various energy homeostasis and adaptive processes in plants (Delgado-Vargas et al. 2000; Bryant et al. 2020). To enable strict but flexible pigment production in a tissue-dependent manner and in response to environmental signals, genes governing plant pigmentation are generally subjected to highly complex transcriptional networks to synthesize proper quantities and qualities of pigments at designated sites and times. For example, the biosynthesis of chlorophylls, green pigments that harvest light energy for photosynthetic electron transport, must be coordinated with chloroplast development because of the phototoxicity of protochlorophyllide, an intermediate in chlorophyll *a* biosynthesis. If protochlorophyllide is mis-accumulated in still-developing tissues where photosynthetic protein complexes are not ready to start photosynthesis, light energy that is absorbed by protochlorophyllide but cannot be utilized for photosynthesis would cause dangerous reactive oxygen species production (Reinbothe et al. 2010; Bryant et al. 2020). Therefore, strictly programmed pigmentation is key to successful plant development and fitness.

GOLDEN2-LIKE (GLK) transcriptional factors are master regulators that govern chloroplast biogenesis in plants (Chen et al. 2016; Hernández-Verdeja and Lundgren 2023). The history of GLK research was initiated with the *Zea mays* (maize) variegated *Golden2* mutant reported a century ago (Jenkins 1926). Cloning of the *Golden2* gene revealed that *Golden2* encodes a transcriptional factor controlling chloroplast development in maize bundle sheath cells (Hall et al. 1998). Since then, its homologs GLKs have been intensively studied in *Arabidopsis thaliana* and many other plants, examining their important roles in chlorophyll biosynthesis and chloroplast development, especially during the greening process. However, studying only GLKs is insufficient for a comprehensive understanding of transcriptional networks behind greening, as many other factors are likely involved in this process.

In this issue of *Plant Physiology*, Han et al. (2023) introduced new players in GLK-mediated chlorophyll biosynthesis and chloroplast development as well as their additional roles in pigment metabolism, hormonal response, and development. *PSEUDO-ETIOLATION IN LIGHT* (*PEL*) genes encode 10- to 15-kDa proteins that belong to an uncharacterized protein family found only in plants and are proposed as a negative regulator of photosynthetic pigment production. Their overexpression downregulated the accumulation of carotenoids in carrots (Iorizzo et al. 2016) and chlorophyll biosynthesis in Arabidopsis (Ichikawa et al. 2006), while its loss-of-function increased chlorophyll content in rice (Zhang et al. 2021). However, due to high PEL redundancy (e.g. 4 isoforms in Arabidopsis), how PEL proteins were involved in the GLK-involving greening step remained unclear. To address this question, Han et al. used CRISPR/Cas9 technology to create a series of multiple knockout mutants, including the quadruple *pel* mutant (*pel1 pel2 pel3 pel4*) and the sextuple *pel glk* mutant (*pel1 pel2 pel3 pel4 glk1 glk2*). The chlorophyll level was elevated in the *pel* mutants at most developmental stages, but this increase in chlorophyll content was not observed in the *pel glk* mutant. This genetic relationship highlights that chlorophyll deficiency in the *glk* mutants was epistatic to the *pel* mutants, suggesting that GLKs act downstream of the PEL proteins in chlorophyll biosynthesis. Inconsistent with the chlorophyll phenotype, the accumulation of anthocyanin, a red/purple antioxidant pigment produced from phenylalanine, was upregulated both in the *pel* mutants and the *pel glk* mutants at a comparable level. This anthocyanin phenotype indicates that the *glk* mutant was not epistatic to the *pel* mutants for anthocyanin accumulation and that the PEL proteins function independently from GLK proteins. Consistently, PEL1 protein physically interacted with the GLK proteins and 6 transcriptional factors associated with anthocyanin accumulation, such as MYB4. In addition to their involvement in such pigment accumulation, PEL proteins play additional roles in determining rosette size, seed size, chloroplast coverage, and abscisic acid–triggered seedling growth inhibition. Collectively, PEL proteins negatively determine pigment contents in GLK-dependent and GLK-independent manners and influence other processes as well (Fig. 1).

**Figure 1.**
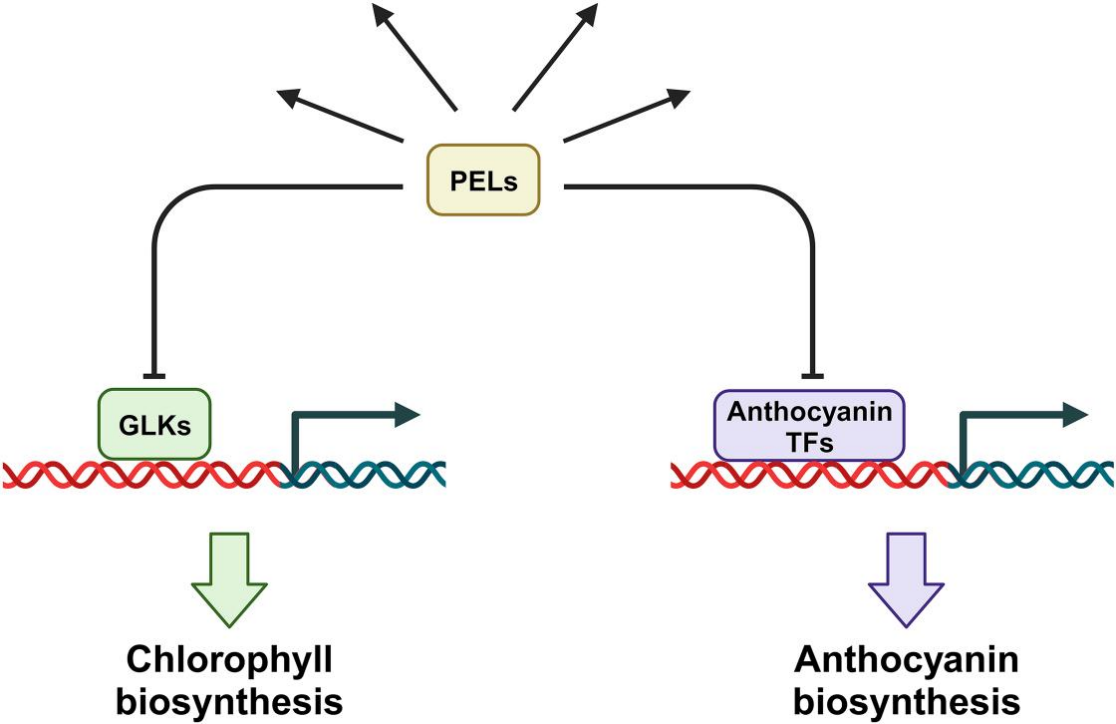
The role of PEL proteins on the biosynthesis of chlorophyll and anthocyanin. PEL proteins separately interact with GLKs and several transcriptional factors (TFs) of anthocyanin production (e.g. MYB4) to negatively regulate chlorophyll and anthocyanin biosyntheses.

The generation and characterization of the high-order multiple *pel* mutants highlight a more general and broader role of PEL proteins in pigmentation processes than previously considered. These findings are not only part of the work toward a comprehensive elucidation of the transcriptional networks that govern the pigmentation process in plants but may also help us to improve crop productivity. GLKs are anticipated to be a promising tool to enhance photosynthesis and increase biomass in model plants and crop species (Hernández-Verdeja and Lundgren 2023; Rosado-Souza et al. 2023). To this end, further investigations are needed to evaluate the effects of PEL proteins on GLK-mediated biological functions to maximize the potential of GLKs. Furthermore, why PEL proteins play such broad roles in plant developmental and adaptation processes is another attractive scientific question to be addressed in the future. Given that they belong to a poorly characterized group of small proteins, their biochemical features may be worthy of further investigation.

## Dive Curated Terms

The following phenotypic, genotypic, and functional terms are of significance to the work described in this paper:

MYB4 Gramene: AT4G38620
MYB4 Araport: AT4G38620

pel1 Gramene: AT4G27650
pel1 Araport: AT4G27650
